# Serum fluoxetine and norfluoxetine levels support the safety of fluoxetine in overdose

**DOI:** 10.1186/s12991-016-0117-z

**Published:** 2016-11-09

**Authors:** Stephanie Pope, Solomon G. Zaraa

**Affiliations:** Division of Child and Adolescent Psychiatry, Department of Psychiatry, University Hospitals Cleveland Medical Center, 10524 Euclid Ave, WO Walker Building, First Floor, Cleveland, OH USA

## Abstract

**Background:**

Previous literature has found fluoxetine to be relatively safe in overdose. This study hopes to examine this idea along with support from published pharmacokinetic information including serum fluoxetine and norfluoxetine levels based on information from a clinical case series.

**Methods:**

Four cases are presented along with vital abnormalities, electrocardiogram abnormalities, and physical exam abnormalities along with amount of overdose and resulting serum fluoxetine and norfluoxetine levels.

**Case Presentation:**

In these four cases, serum fluoxetine and norfluoxetine days after overdose were found to be in a range believed to be within the treatment range. No abnormalities were found on electrocardiogram but some patients (3) were found to have slight elevations in heart rate.

**Conclusion:**

Fluoxetine is relatively safe in overdose. This study supports previous literature. Future directives for research can be directed towards when serotonergic, including fluoxetine, medications can be introduced or restarted in patients who have overdosed. Research could also focus on if the introduction of another medication, such as carbamazepine, to induce metabolism of a medication, such as fluoxetine, after an overdose.

## Background

Previous research has provided ample evidence to conclude fluoxetine without tricyclic antidepressants as relatively safe in overdose. Early literature found sinus tachycardia, convulsions, depressed ST segments on electrocardiogram (ECG), elevated diastolic blood pressure, drowsiness, and agitation in a series of case reports and chart reviews of fluoxetine in overdose [1–5]. A larger chart review of 234 cases was completed including 20 pediatrics patients and 67 patients who ingested fluoxetine alone. In this chart review, the 20 pediatric patients ranged in age between 10 months and 4 years old. The mean dose ingested was 23.4 mg or 1.76 mg/kg. Of the 20 pediatric patients, 18 remained asymptomatic, while hyperactivity and diarrhea were reported in a 2-year-old and sleepiness was reported in a 23-month old. Of the 67 adults in this study, the mean dose ingested was 544 mg, while 30 were asymptomatic, 15 were found to have tachycardia, 14 reported drowsiness, five with tremor, four with vomiting and four with nausea, one with euphoria, one with headache, one with sore throat, one with trigeminy, one with junctional rhythm, and one with abdominal pain [6].

Meanwhile, serum concentrations of fluoxetine and its active demethylated metabolite, norfluoxetine, have been studied. One study found four patients were treated with 80 mg/day for 52 ± 8 weeks and had their serum measured for fluoxetine and norfluoxetine while taking the medication and then 4 and 8 weeks after discontinuation. Mean fluoxetine and norfluoxetine levels during treatment were 620 ± 49 ng/ml and 496 ± 49 ng/ml, respectively. After 4 weeks of discontinuation, mean fluoxetine and norfluoxetine levels during treatment were 55 ± 19 ng/ml and 184 ± 40 ng/ml, respectively. After 8 weeks of discontinuation, fluoxetine and norfluoxetine levels during treatment were 0 ng/ml and 47 ± 20 ng/ml, respectively. Age and sex of the patient did not impact metabolism of this study [7]. Two other studies found sex did impact serum concentrations and metabolisms. In a study of 10–17 year olds [8] and adults [9], it was found that males had lower fluoxetine and norfluoxetine serum levels similar to comparing Case 1 and Case 2 although admitting vastly less sophisticated.

Unfortunately, Patients have continued to overdose with fluoxetine. The purpose of this study is to examine serum fluoxetine and norfluoxetine levels as a product of time from fluoxetine overdose in relationship to their clinical presentations. The goal of this study is to address the gap of knowledge and complications from fluoxetine overdose in clinical cases. The hypothesis of this study is that fluoxetine is relatively safe in overdose.

## Methods

Participants were identified by the authors as minors admitted to an acute psychiatric unit for fluoxetine overdose between January 1, 2011 and April 1, 2015. Pregnant patients were excluded. Once patients were identified, their charts were reviewed and data such as age, sex, vitals, serum fluoxetine and norfluoxetine levels, electrocardiograms and physical exam findings, and psychiatric medication administered during hospitalization after the reported intentional overdose were extracted from the chart. This information was then correlated to existing literature, specifically plasma concentrations of fluoxetine and norfluoxetine.

This study was approved by the affiliated hospitals IRB board.

## Cases

### Case 1

17-year-old female admitted for an intentional overdose of 120 mg of Fluoxetine. She was found two days after her ingestion to be diffusely hyper-reflexic and this was thought to be due to an elevation in serotonin. She was restarted on her outpatient regimen of fluoxetine 30 mg 5 days after her ingestion. After approximately 144 h from her ingestion and one administration of her outpatient dose the day prior, her serum fluoxetine level was 139 ng/mL and her serum norfluoxetine level was 205 ng/mL.

### Case 2

16-year-old male admitted for an intentional overdose of 680 mg of Fluoxetine with 10 unknown dose tablets of acetaminophen. After approximately 148 h from his ingestion, his serum fluoxetine level was 226 ng/mL and his serum norfluoxetine level was 205 ng/mL. He was initially admitted to the intensive care unit and monitored for transaminitis which he ultimately did not suffer from. He was transferred to the acute inpatient psychiatric unit. Considering these levels, carbamazepine was discontinued and he was started on escitalopram targeting depression.

### Case 3

14-year-old female admitted for an intentional overdose of an unknown amount of fluoxetine with phenylephrine, multivitamins, dextromethorphan, glucosamine, chlorpheniramine, and acetaminophen. She was obtunded on admission, given charcoal to induce vomiting and ultimately intubated. She was febrile and found to have aspiration pneumonia. Approximately 163 h after her ingestion, her serum fluoxetine level was 464 ng/mL and her serum norfluoxetine level was 453 ng/mL. Another blood draw 211 h after ingestion found her serum fluoxetine level to be 163 ng/mL and her serum norfluoxetine level to be 468 ng/mL. Once she was medically well, she was transferred in an acute psychiatric unit. After her second serum fluoxetine draw, she was restarted on half of her outpatient dose of 40 mg PO daily for a day and then increased the next day to her outpatient dose of 80 mg daily.

### Case 4

15-year-old female admitted after an intentional overdose of 200 mg of fluoxetine with 30 mg of melatonin. Her serum fluoxetine was found to be 272 ng/mL and norfluoxetine to be 80 ng/mL about 67 h after ingestion. She was started on escitalopram 20 mg daily, the day after her serum blood drawn.

## Results

Results are listed in Tables 1 and 2.

**Table 1:** CASES including demographic information, serum levels, and time after ingestion serum were drawn

Age/sex	BMI	Overdose (mg)	1st level of F (ng/mL)	1st level of Norf (ng/mL)	Time after ingestion (hours) for 1st level	2nd measured level of Fluoxetine	2nd measured level of NorF	Time after ingestion (hours) for 2nd level
17F	28.4	240	139	205	144	Not completed	Not completed	Not completed
16M	21.1	680^a^	226	255	148	37	165	316
14F	20.0	Unknown^a^	464	453	163	338	468	211
15F	21.6	20^a^	272	80	67	Not completed	Not completed	Not completed

*F* serum fluoxetine; *NorF* serum norfluoxetine

^a^Fluoxetine overdose with other medications in overdose

**Table 2:** CASES including ECG, vital and physical exam abnormalities

Age/sex	ECG ab	Vital ab	PE ab
17F	None	HR = 103 on HD #4	Hyperreflexia on the second day of hospitalization
16M	None	HR = 105 on presentation	None
14F	None	Febrile due to pneumonia	Obtunded on presentation
15F	None	HR = 102 on presentation	None

HR > 100 considered abnormal

Diastolic blood pressure > 100 considered abnormal

*HR* heart rate; *HD* hospital day; *Ab* abnormalities; *ECG* electrocardiogram; *PE ab* Physical exam abnormalities

## Discussion

In all four of these cases, the levels found after an intentional overdose (between 67 and 163 h later) were within levels considered therapeutic. Limitations in this study include the subjective matter of reporting an intentional overdose, amount and nature of the overdose and time of reports ingestion, and potential errors in documenting such information. Another limitation in this study includes the exclusion of CYP450 information. Future directives could include clinical correlations of safety compared to serum levels and CYP450 activities.

Vital abnormalities found included a heart rate slightly over 100. None of the patients had ECG abnormalities or elevations in diastolic blood pressure as noted by previous studies. One patient was febrile but this was most likely related to aspiration pneumonia. The majority (three out of four) of these cases involved an ingestion of fluoxetine and other agents, which may complicate the pharmacokinetics and serum levels studied here.

The pharmacokinetics of fluoxetine and norfluoxetine is dependent on its administration either as an acute overdose or chronic therapy requiring weeks to reach stead state [7, 10–12]. This is beyond the scope of this study as administration prior to overdose was not collected in the chart review.

Interestingly enough, Case 3, admittedly the most medical complicated in this series, was noted to have an increase in her serum norfluoxetine levels between the first and second blood draw. This could be related to a phenomenon found in a case of massive quetiapine overdose in which serum concentration levels did not follow first-order kinetics. This published case suggests that the initial concentration was related to quetiapine being distributed within tissues and slower redistribution from tissues to serum prior to hepatic metabolisms [13].

## Conclusions

This study supports previous literature stating that fluoxetine is relatively safe in overdose. Vitals, ECGs, and serum fluoxetine and norfluoxetine levels can be monitored. Further research can be directed toward when serotonergic, including fluoxetine, medications can be introduced or restarted in patients who have overdosed.

## Abbreviations

ECG: electrocardiogram
mg: milligram
mg/kg: milligram per kilogram
ng/ml: nanogram per milliliter
